# A Teledermatology Pilot Programme for the Management of Skin Diseases in Primary Health Care Centres: Experiences from a Resource-Limited Country (Mali, West Africa)

**DOI:** 10.3390/tropicalmed3030088

**Published:** 2018-08-17

**Authors:** Ousmane Faye, Cheick Oumar Bagayoko, Adama Dicko, Lamissa Cissé, Siritio Berthé, Bekaye Traoré, Youssouf Fofana, Mahamoudan Niang, Seydou Tidiane Traoré, Yamoussa Karabinta, Mamadou Gassama, Binta Guindo, Alimata Keita, Koreissi Tall, Somita Keita, Antoine Geissbuhler, Antoine Mahé, Teledermali Team

**Affiliations:** 1 Department of Dermatology, Faculty of Medicine and Odontostomatology, Bamako, Mali; adadicko@yahoo.fr (A.D.); lamissa05@gmail.com (L.C.); siritio_b@yahoo.fr (S.B.); b_traore@ymail.com (B.T.); youssouffofana346@yahoo.fr (Y.F.); ykarabinta@yahoo.com (Y.K.); gasdiaby@yahoo.fr (M.G.); binta.guindo@yahoo.fr (B.G.); alimatakeita@yahoo.fr (A.K.); koreissit@yahoo.fr (K.T.); somitak@yahoo.fr (S.K.); 2 CERTES, Bamako, Mali; cob281@yahoo.fr (C.O.B.); mniang@certesmali.org (M.N.); trawore@keneya.net (S.T.T.); 3 Service de Dermatologie, Hôpital Pasteur, Colmar 68000, France; antoine.mahe@ch-colmar.fr; 4 Département de Radiologie et Informatique médicale, Université de Genève, Genaven 1211, Switzerland; antoine.Geissbuhler@hcuge.ch

**Keywords:** teledermatology, Africa, primary health care, skin diseases, tele-expertise

## Abstract

In sub-Saharan Africa, in particular in rural areas, patients have limited access to doctors with specialist skills in skin diseases. To address this issue, a teledermatology pilot programme focused on primary health centres was set up in Mali. This study was aimed at investigating the feasibility of this programme and its impact on the management of skin diseases. The programme was based on the store-and-forward model. Health care providers from 10 primary centres were trained to manage common skin diseases, to capture images of skin lesions, and to use an e-platform to post all cases beyond their expertise for dermatologists in order to obtain diagnosis and treatment recommendations. After training, the cases of 180 patients were posted by trained health workers on the platform. Ninety-six per cent of these patients were properly managed via the responses given by dermatologists. The mean time to receive the expert’s response was 32 h (range: 13 min to 20 days). Analysis of all diseases diagnosed via the platform revealed a wide range of skin disorders. Our initiative hugely improved the management of all skin diseases in the targeted health centres. In developing countries, Internet accessibility and connection quality represent the main challenges when conducting teledermatology programmes.

## 1. Introduction

To date, there is a general agreement that skin diseases (SDs) should be considered a public issue, in particular in developing countries [1] where their management is subject to several challenges. Prevalence studies from different continents have pointed to a high prevalence of SDs, especially in children, at an average of 30% (range 6 to 87%) [2,3,4,5,6,7,8,9,10]. While SDs are the fourth leading cause of consultations in health centres [11,12], patients have limited access to skin doctors with specialist skills in skin disease; these generally prefer to settle in big cities. In Africa, the situation is critical; the ratio of dermatologists to the population is extremely low, and ranges from 1 for every 500,000 to 1 million inhabitants. Mali, a country of 17 million inhabitants, had only 10 dermatologists in 2015; all were posted in the capital city. The pressure for skin care is mainly borne by front-line health facilities, and most of those personnel are swamped with other health priority programmes (immunization, malaria, tuberculosis, HIV, etc.). The capacity of such agents to manage common SDs and leprosy has been questioned [13]. The overall context of care provision in developing countries represents an additional challenge. Warm climate and overcrowded settings are likely to foster the skin infections that represent almost 70% of the skin disorders that motivate consultations [14]. Hitherto, there are no international guidelines or treatment recommendations, with the exception of leprosy, a disease that has been eliminated in most endemic countries. A previous study [15] identified the effectiveness of one-day training to improve the management of common SDs in primary health care centres. The issue remains as to how the remaining cases that need the expertise of a specialist dermatologist can be managed. The recent boom of information and communication technologies (ICTs) and their use in medicine, particularly dermatology (teledermatology), have transformed the way in which skin disorders can be properly managed in remote areas. By the year 2013, more than 560 papers were published on this subject [16] and 2000 patients were evaluated, with good kappa concordance test values ranging from 0.63 to 0.95 [17,18,19,20,21]. This growing awareness for teledermatology prompted us to take advantage of this technology to improve skin care in Mali through a pilot programme. 

The aim of this study was to test the feasibility and the impact of a teledermatology programme on the management of skin diseases, at primary health care level.

## 2. Methods

### 2.1. Ethical Statements

The study protocol was approved by the Institutional Review Board of the National Ethical Committee for Health and Life Sciences of Mali (*Comité National d’éthique pour la santé et les sciences de la vie, CNESS*). Prior to the study, informed consent was obtained from adult participants andfrom the parents or legal guardians of minors after detailed explanation of the study protocol. In accordance with the ethical review committee requirements, patient information was made confidential. Our intervention was only focused on the health system and the management of patient care so that this could be improved in Mali. There was no new drug tested nor blood test performed in the study.

### 2.2. Health Care System in Mali

In Mali, as in many African countries, the health care system in the public sector comprises three levels of care. The first level, also called primary health care level, comprises community health centres (*centre de santé communautaire*) and district referral health care centres (*centre de santé de référence*) where the first-level administration (district) is sited. Regional hospitals and regional health administrations represent the second health care level (region). The capital city hosts the third health care level, represented by teaching hospitals and the national health administration, where the national health policy is elaborated. In 2015, the country had 5 teaching hospitals, 8 regional hospitals covering 10 health regions, 63 district referral centres, and 1215 community health centres. The present study was focused on this last group, the primary health care centres that represent the entry point to the health system where health care is delivered by general physicians, nurses, and in a few cases, minimally trained health care workers (HCWs).

### 2.3. Study Design, Sites, and Duration 

**Figure 1:**
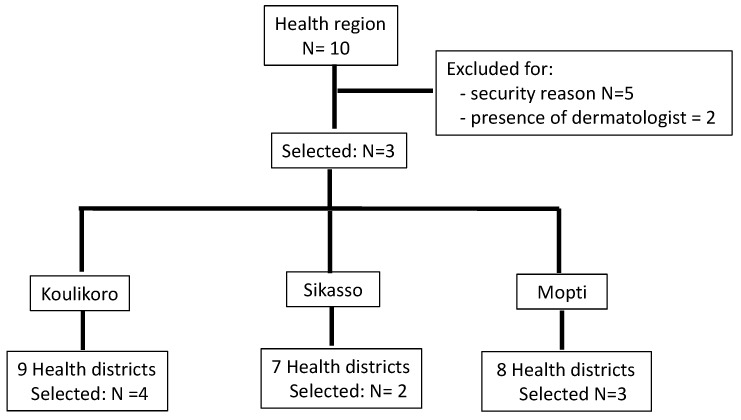
Flow chart of the selection of study sites: health region and districts.

From July 2015 to February 2017, we carried out a pilot teledermatology programme based on the store-and-forward (SAF) model, focused on front-line health care delivery services. Of the 10 health regions in Mali, we selected three regions for the purpose (Koulikoro, Sikasso, and Mopti); the rest were excluded for either security reasons or the close presence of a dermatologist. In each health region, selected three health districts were also selected: the administrative centre of the health region plus two or three more health districts randomly selected thereafter, depending on the size of the region (Figure 1). Overall, 9 health districts were involved in the study planned to last for 18 months: 6 months for pre and post programme evaluation and training, and 12 months for utilization of the system. 

### 2.4. Intervention—Equipment and Participants

The first step of our intervention was training. In each health centre, two health care workers were invited to participate in the training session which was held in the main dermatological ward, at the Marchoux Institute in the capital city Bamako where expert dermatologists were posted. During three consecutive days, the selected HCWs were respectively given information on the algorithmic approach for the management of common skin diseases identified in the pilot project [15], the use of a digital camera, and computer skills including type writing, the use of Internet (email), and the tele-expertise platform named ‘*Bogou*’ [22] (that means mutual assistance or help in all situations in the Djerma and Songhoï languages spoken in Mali, Niger, and Cameroon). This e-platform is a web-based application with secured access through a personal login and password [23]. It was developed with Java to run on several operating systems and work reliably with unstable and low-bandwidth connections. All data in *Bogou* are encrypted using a public and private key system [23]. Dermatological training was focused on common SDs, i.e pyoderma, superficial mycoses including tinea capitis, scabies, and conditions with pale patches inclusive of leprosy; attendees were encouraged to refer via the platform all skin disorders assessed as being beyond their competency and any case that did not fit with the algorithm. Theoretical training was also completed with clinical case studies and practical exercises. At the end of the training session, participants were issued with a booklet addressing the management of common SDs as cited above, a digital camera, and a kit for Internet connection including a 3G key USB.

### 2.5. Teledermatology Operating Procedures

In practice, when encountering a case that needed a consultation with a dermatologist, the health agent captures one or more pictures with a digital camera, logs on to a computer, uploads the images from the camera, connects to the platform, and uploads the pictures with a short medical history of the patient. The dermatologist (expert) immediately receives an email alert indicating an assignment for a case for diagnosis that has been sent. The expert also will log onto the platform, analyse the images, and send back concise details on the disorders and treatment recommendations. At any time, all participating HCWs could access pictures and responses. Importantly, the workload involved in responding to the HCWs relied mainly on one individual (OF) backed by a second in case the first one was not available; only one response to each request was given. The validity of all diagnoses and management strategies given was checked by another dermatologist based in France, who had expertise in tropical dermatology (AM). Clinical supervision was performed quarterly by a team (dermatologist and computer scientists) to confirm difficult diagnoses through skin biopsy and to solve technical issues.

### 2.6. Evaluation

We planned to evaluate the feasibility of the study and its impact on the management of common SD. While implementing and performing the programme, all relevant issues and challenges were recorded. The following indicators were also evaluated: total number of store-and-forward teledermatology consults via the *Bogou* platform, the number of cases that received a clear diagnosis and treatment, the turnaround time to receive the response from experts, the pattern of SDs referred to experts, the diagnosis concordance between experts, and the proportion and pattern of SDs as seen by HCWs before and after intervention. All SDs diagnosed via teledermatology were also compared to those seen in a dermatological setting where experts were working over the same period of time. Patient and care provider feedback was also assessed to measure the level of satisfaction and acceptance of the system.

### 2.7. Data Collection and Statistical Analysis

A standardized questionnaire was used to collect the data from patients who visited the targeted health centres for skin diseases and patients managed via the system. The opinions of both HCWs and the patients were also collected and then sorted by level of satisfaction. The software SPSS.16.0 (IBM Company, Chicago, IL, USA) was used for data capture and analysis. The chi-squared test was used to compare two proportions.

## 3. Results

### 3.1. Characteristics of the Study Sites and Trainees:

Overall, 20 HCW from 9 health centres were involved in the study: 9 nurses and 11 general physicians. Before the study, 6 out of 20 HCW had never touched a computer and had no email address (nurses only). Of the 9 health centres, only 4 had an Internet connection (Table 1). Not a single centre had a digital camera.

### 3.2. Skin Diseases Diagnosed by HCWs

Before the intervention, the proportion of dermatological consultation varied from 8% (Ngolobougou, 51/612) to 65% (Sikasso, 692/1067) with a mean of 20%. After the training session, these proportions were respectively 9% and 63% (Table 1). There was a slight increase in the dermatological activity of all centres, but the difference was not significant.

The pre-programme evaluation revealed that the five top diagnoses of SDs retrieved in the health centre logbook were: wounds (10.8%), allergies (8.8%), mycosis (5%), genital infection (7.3%), and pruritus (4.7%) (Table 2). At the post-programme evaluation, pyoderma (28.4%), eczema (13.5%), dermatophytosis (6.9%), tinea capitis (5.3%), and prurigo (4.4%) were the most common SDs. There was a huge decrease in the proportion of patients with unclear diagnosis, which decreased from 53.7% (322/599) to 12.4% (84/679) before and after training, respectively (*p* < 10^−6^). Clearer diagnoses were also observed during the post-programme period.

### 3.3. Management of Skin Diseases via Teledermatology

**Figure 2:**
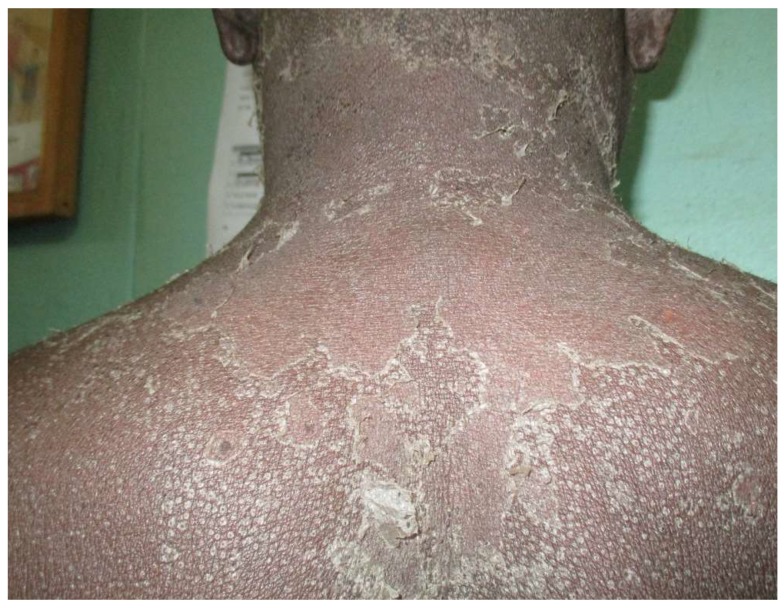
Drug eruption caused by amoxicillin intake following dental extraction.

**Figure 3:**
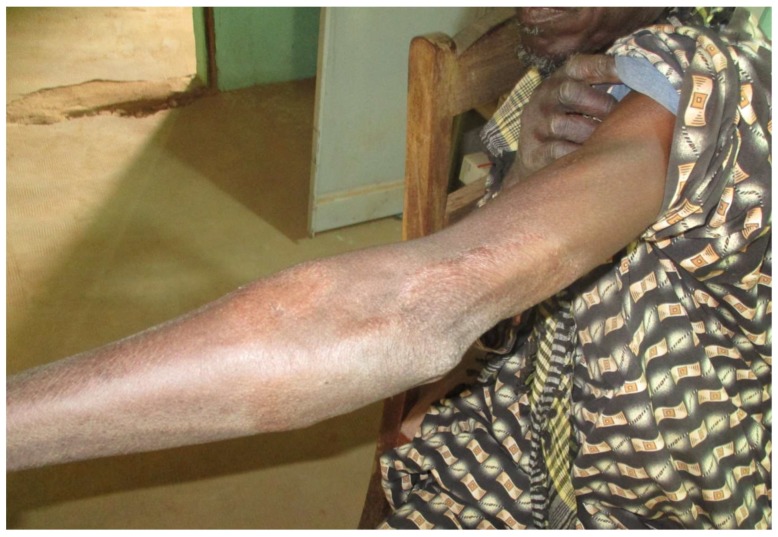
Paucibacillary leprosy: note the large hypochromic patch of the arm.

**Figure 4:**
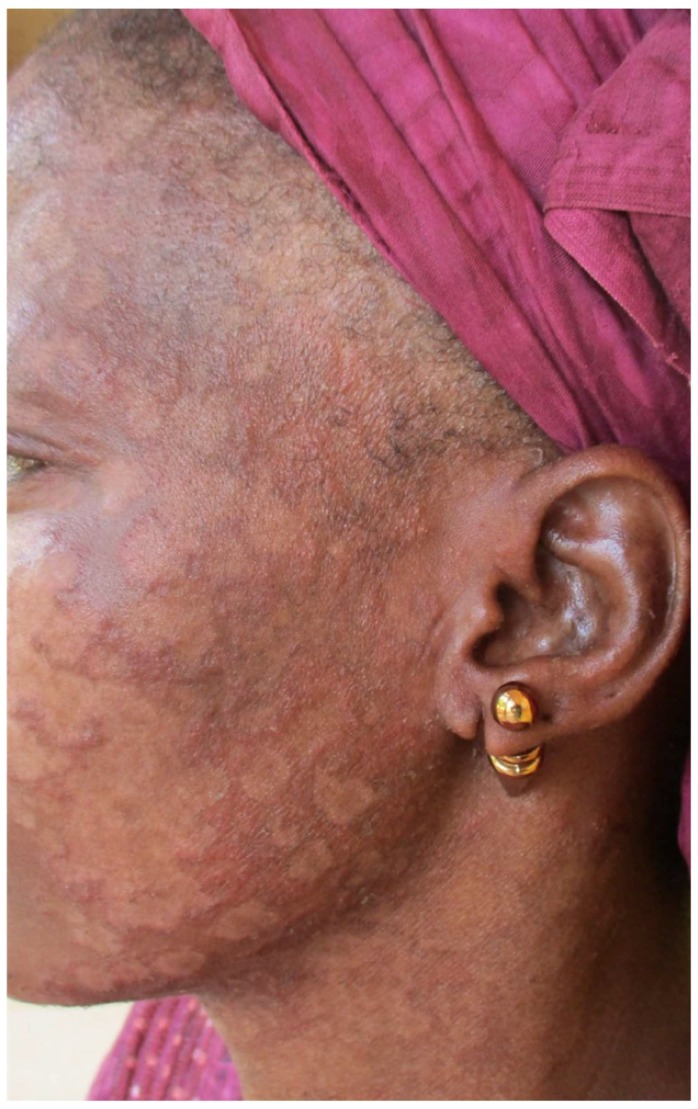
Tinea facei in women using skin bleaching creams (clobetasol and hydroquinone).

**Figure 5:**
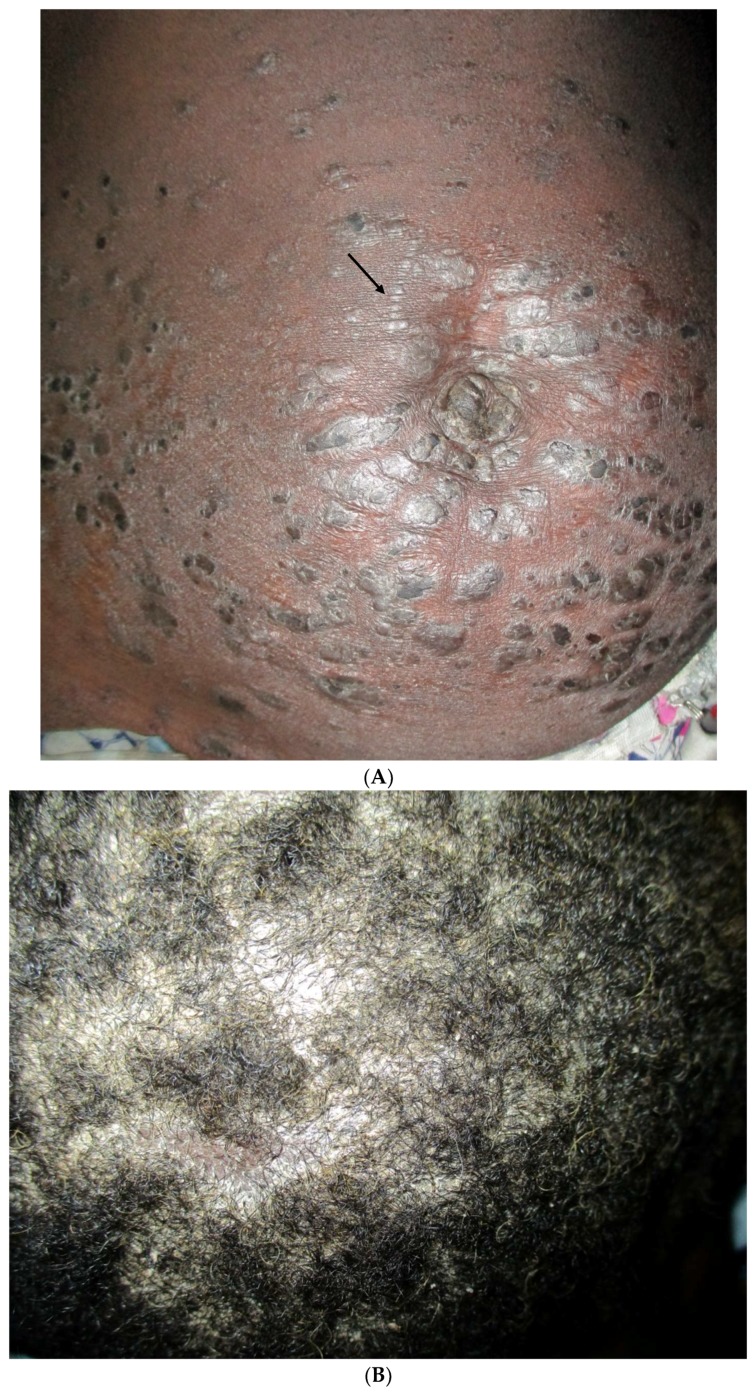
(**A**) Psoriasis in a 35-year-old woman: erythematous papules and plaque over the abdomen. Note the Köbner phenomenon; (**B**) Scalp involvement of the patient.

This started immediately after training and continued over one year, from October 2015 to October 2016. Overall, 180 patients who consulted at the targeted health facilities were diagnosed and treated via our platform. The mean number of patients managed per month was 15, with a range of 5 (January) to 24 (August). Regarding the centre activity, the number of patients seen ranged from 3 patients (Nara) to 36 patients (Banamba) (Table 1). The number of images sent per patient varied from 0 to 13, with an average of 3.6. All patients except one accepted being photographed. The average turnaround time to receive the expert’s response was 32 h (range from 13 min to 20 days). Except in two cases referred to the dermatological ward in the capital city (one case of bullous pemphigoid and one case of psoriasis) all patients were managed locally. Analysis of diseases diagnosed via the platform illustrated a wide range of conditions (Table 2) (Figure 2, Figure 3, Figure 4 and Figure 5). The five top diagnoses were: eczema (25/180), dermatophytosis (22/180), pyoderma (10/180), prurigo (11/180), and psoriasis (7/180). However, other conditions such as keratoderma plantaris, vitiligo, small pox, pemghigus, drug-related eruption, and psoriasis were also seen. Miscellaneous diseases included lupus erythematosus (2 cases), leprosy (1 case), transient pustular melanosis (1 case), haemangioma (1 case), epidermal cyst (1 case), syringoma (1 case), and alopecia areata (1 case) (Table 3). The overall diagnosis concordance between dermatologists involved in the programme was 95% amongst the 40 cases randomly selected. Experts had divergent diagnoses in two cases in which the quality of the images was judged unsuitable for correct diagnosis. Importantly, clinical supervision of the health centres enabled the diagnosis of five patients through skin biopsy: two cases of psoriasis and one case each of pemphigus, lichen planus, and epidermal cyst, respectively. The quality of information and photographs given to experts was considered not good enough for diagnosis in six patients (3%). In addition, we failed to diagnose five more cases. Overall, 11 patients had no diagnosis.

**Figure 6:**
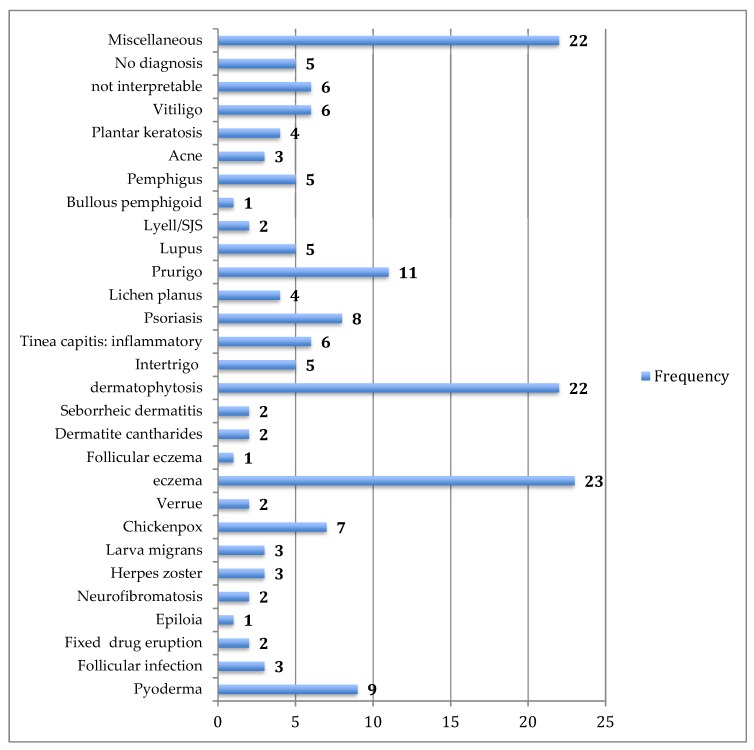
Pattern of skin diseases diagnosed via the platform in 180 cases posted by primary health care workers.

**Figure 7:**
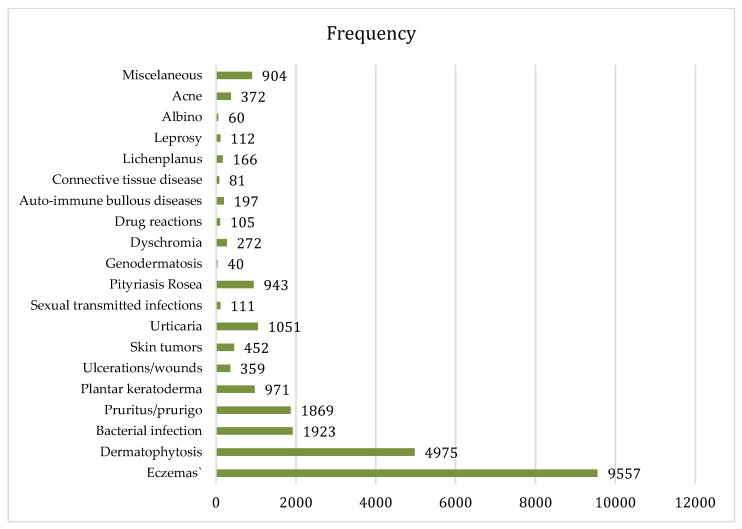
Pattern of skin diseases seen in the Referral centre of Dermatology at the Marchoux Institute in 2015: 24,520 cases.

In the dermatology clinic of the capital city, 24,520 patients were consulted during the study period. The five top diagnoses observed in these patients were: eczema, dermatophytosis, bacterial infection and prurigo, or pruritus and plantar keratoderma. This pattern was similar to that of cases managed via teledermatology during the same period (Figure 6 and Figure 7).

### 3.4. Satisfaction of HWCs and Patients

Interviews regarding the level of satisfaction involved 10 health providers and 37 patients managed via the system. Eight out of 10 HCWs declared that they were satisfied by the management of patients via the platform, this included four who were strongly satisfied. The time to receive the response from the expert was considered to be reasonable for seven out of 10 HCWs and response times for two were ‘moderately’ acceptable. According to some health care providers, our intervention enhanced both health centres and general health care practices. They stated: “I felt more confident when facing a patient with SD”; or: “presently, people look at me differently (with more attention) when compared to the time before the programme started”. They also though that some programme aspects could be improved: the time to receive the response from the expert, and the platform. The concept of an android version for mobile phones and improved quality of the Internet connection in each region were considered relevant.

All the 37 patients interviewed stated that they were strongly satisfied and would recommend teledermatology to other patients. The fact that they were locally managed was very much appreciated. They also expressed the need to sustain this initiative.

## 4. Discussion

This study was a teledermatology pilot programme based on a store-and-forward model and designed to work at a primary health care level in a developing country. Its implementation and evaluation definitely validated the feasibility of a teledermatology programme in a resource-limited area. The usefulness and challenges of such a programme has also been highlighted. There was a huge improvement in the management of all skin diseases in the targeted primary centres, for both common skin diseases and those beyond the expertise of the HCWs. A large number skin diseases were identified and managed locally by health care workers whose knowledge was also improved as long as the intervention continued. The equipment used for connection in this study was very simple, low-cost, and suitable when compared to a live interactive model that requires more equipment and greater technological expertise.

However, several limitations should be addressed. These include the selection of only three health regions, the small number of randomly selected health centres as compared to the total number of health centres in Mali, and the small number of teledermatology requests from one centre (Banamba) due to either the quality of the Internet connection or the lack of HCW commitment to ask for help.

Several papers have addressed the usefulness and effectiveness of teledermatology programmes [16,24]. The greatest number of published studies were performed in the United States, followed by the United Kingdom, Spain, the Netherlands, Italy, and Austria [25] Very few studies were conducted in Africa [26] or in sites such as prisons with greater health care needs [27]. As used in our study, store-and-forward continued to be the most common delivery modality [28]. The present study helps to bridge the gap of health care quality between front line health facilities and dermatologic wards. In rural areas where is a high prevalence of SDs and lack of specialists the needs of care for many people are also met.

The post-programme evaluation of health centres revealed that the management of common SDs was improved as shown by the decrease in the proportion of patients with either unclear diagnosis or ‘dermatoses’ (allergy, mycosis), and an increase in the number of clear diagnoses, i.e. pyoderma, dermatophytosis, scabies, and eczema. In the pre-programme evaluation, not a single case of very common disorders such as prurigo, urticaria, chickenpox, and miliaria was recorded in the centre logbook. After training, there was a huge improvement in the management of these disorders due to the dermatologic training. Along with this management shift, the skills of HCWs regarding computer science were also improved. One of our HCWs, who had never touched a computer, sent 17 requests for tele-expertise. In addition, two health centres were completely run by nurses, who were beginners in terms of computer science and the Internet. While no case of prurigo was mentioned in the pre-programme evaluation, many cases were noticed after intervention. This improvement could be in part related to the training module of common SDs to which participants have been exposed. It also confirmed the effectiveness of one-day training of primary HCWs in the management of common SDs with an algorithmic approach [15] that was likely to have helped to control the number of requests for teledermatology and avoided experts being swamped by HCW requests. We assume that the trained health staff managed many skin disorders themselves and e-referred only those for which dermatological expertise was required; this indicates that the programme expectations were met. The improvement in the diagnosis of SD in the targeted health centres as shown in the post-intervention evaluation might also have been a result of the continuing medical education created by the regularly-sent responses of experts via the e-platform that all HCW had the possibility to see and learn from. The clinical supervision by dermatologist confirmed cases in which the diagnosis was uncertain. The similarity of leading causes for visits in both primary and specialized health centres, as shown in this study, indicates that the pattern of SDs is not related to the level of health care facilities, but to the epidemiology of SDs. It also points out the necessity to set up and to sustain such initiatives, particularly in areas with poor geographic accessibility and weak coverage by dermatologists.

Despite the profound penetration of information and communication technology in Africa, the actual functioning of such tools has been repeatedly questioned given the frequent shortages of electric power, maintenance issues, and the low quality of connectivity. This prompted us to choose low-level technology with an adapted tool that comprised a 3G key connection and digital camera, as computers were available in all centres. This equipment worked well, and more than 90% of patients were properly managed. It should be underlined that the estimated cost of the teledermatology kit tool can be can easily covered by the cost of referral of one patient from Mopti to Bamako, the capital city. The mobile version of *Bogou*, which has been recently put into production, will lead in the future to a considerable drop in equipment costs. Indeed, almost every HWC has a smartphone in Mali.

Some issues not addressed in this study should be addressed further, in particular in developing countries when setting up a teledermatology programme. These include privacy issues as well as ethical and medicolegal responsibility in cases of injury and unsolved cases.

## 5. Conclusions

The implementation of a teledermatology programme based on a store-and-forward process in primary health care services is feasible and can positively impact the management of skin disorders. Development and testing of specific and secure applications for the e-management of skin disorders should be promoted. The expansion of such programmes is appealing in resource-limited countries where there are financial constraints and dermatologists are lacking.

## Figures and Tables

**Table 1:** Evaluation of health centres: proportion of skin diseases before and after training and distribution of trainees and cases posted via the platform.

Health Centre	Number of Trainees	Number of Cases Posted via the Platform	Before Training		After Training	
			Number of Skin Diseases (%)	Number of Consults	Number of Skin Diseases (%)	Number of Consults
Koulikoro ^a^	2	9	82	900	76	756
Banamba ^a^	3	32	185	1512	185	1426
Nara	2	3	95	542	85	501
Sikasso ^a^	2	16	692 (65)	1067	706 (63)	1121
Ngolobougou	1	36	51 (8)	612	65 (9)	723
Kadiolo	2	6	60	415	79	608
Mopti ^a^	4	35 ^b^	108	902	166	1091
Douentza	2	17 ^b^	45	480	60	461
Bankass	2	26	78	678	106	816
TOTAL	20	180	1396	7108	1528	7503

^a^ Centres where the Internet connection was available before teledermatology intervention; ^b^ centre exclusively run by a nurse.

**Table 2:** Pattern of skin diseases observed in health centres before and after intervention: report on the six-month period.

Skin Diseases	Before Intervention	After Intervention
Dermatosis	322	84
Eczema	6	92
Dermatophytosis	-	47
Mycoses	30	7
Tinea capitis	3	36
Pyoderma	16	193
Wounds	65	-
Urticaria	-	11
Allergy	53	14
Follicular infection	8	-
Pruritus	28	-
Scabies	3	15
Onychodystrophy	-	13
Candidiasis	-	20
Measles	-	12
Herpes zoster	-	3
Herpes labialis	-	5
Genital infection	44	8
Prurigo		30
Erysipelas	7	11
Rash	4	8
Molluscum contagiosum	-	3
Aphthous ulcers	-	3
Patches	6	-
Intertrigo	-	6
Chickenpox	-	20
Vitiligo	-	4
Lipoma	-	1
Miliaria	-	22
Burn	4	-
Keloid	-	4
Ulceration	-	7
**Total**	**599**	**679**

**Table 3:** Miscellaneous skin conditions diagnosed via the platform in 22 cases out of 180 cases posted from health centres.

Skin Disorders	Frequency
Sun burn	2
Eczematised miliaria	2
Hemangioma	1
Alopecia areata	2
Epidermal cyst	1
Vulvovaginitis	1
Leprosy	1
Viral exanthema	1
Multiple keloid	2
Transient pustular melanosis	1
Onychodystrophy	1
Eruptive hidradenoma	1
Lichen simplex	1
Plantar ulcer	1
Congenital keratoderma	2
Varicose veins	2
**Total**	**22**
